# Development of a centrifugation-based method for the qualitative molecular detection of *Cryptosporidium* spp., *Giardia duodenalis* and *Enterocytozoon bieneusi* in surface water samples

**DOI:** 10.1016/j.mex.2026.104195

**Published:** 2026-10-08

**Authors:** Susanna Adelaide Sechi, Chiara Stocchero, Carolina Allievi, Diego Copetti, Fabrizio Stefani, Lucia Valsecchi, Alessandra Cafiso, Chiara Bazzocchi, Maria Teresa Manfredi

**Affiliations:** a Department of Veterinary Medicine and Animal Sciences, Università degli Studi di Milano, Lodi, Italy; b Research Laboratory of Animal Parasitic Diseases and Zoonoses (ParVetLab), Università degli Studi di Milano, Lodi, Italy; c National Research Council of Italy, Water Research Institute (CNR-IRSA), Brugherio Unit, Brugherio, Italy

**Keywords:** Surface water, Waterborne pathogens, Environmental surveillance, DNA detection, Centrifugation

## Abstract

Workflow for waterborne parasites DNA detection in surface water.

Screening for environmental surveillance in monitoring and resource-limited settings.

Zoonotic protozoa and microsporidia are waterborne pathogens with implications for animal and human health. Their resistant stages can persist in freshwater. Standard reference methods for water analysis are often limited to specific parasites, require large water volumes, and depends on expensive equipment. Practical alternatives are needed to facilitate parasite detection in water. This study aimed to develop a pellet-based workflow to allow the DNA detection of *Cryptosporidium* spp., *Giardia duodenalis* and *Enterocytozoon bieneusi* from the same surface water sample. Three organism-specific spiked controls and eight environmental samples from four freshwater sites, collected over two months, were processed by centrifugation to concentrate the pellet fraction, while the supernatant recovered post-centrifugation was filtered through membranes. The samples were collected as part of a broader ongoing year-long monitoring project. DNA was extracted from both fractions and analysed by qualitative PCR assays. Pellets yielded positive results whereas no positivity was detected in filter membranes, suggesting pellet fractions as the most suitable for downstream molecular analysis. This qualitative pellet-based processing approach was designed to provide an assessment and a practical screening strategy for environmental surveillance.

## Graphical abstract

**Figure:**
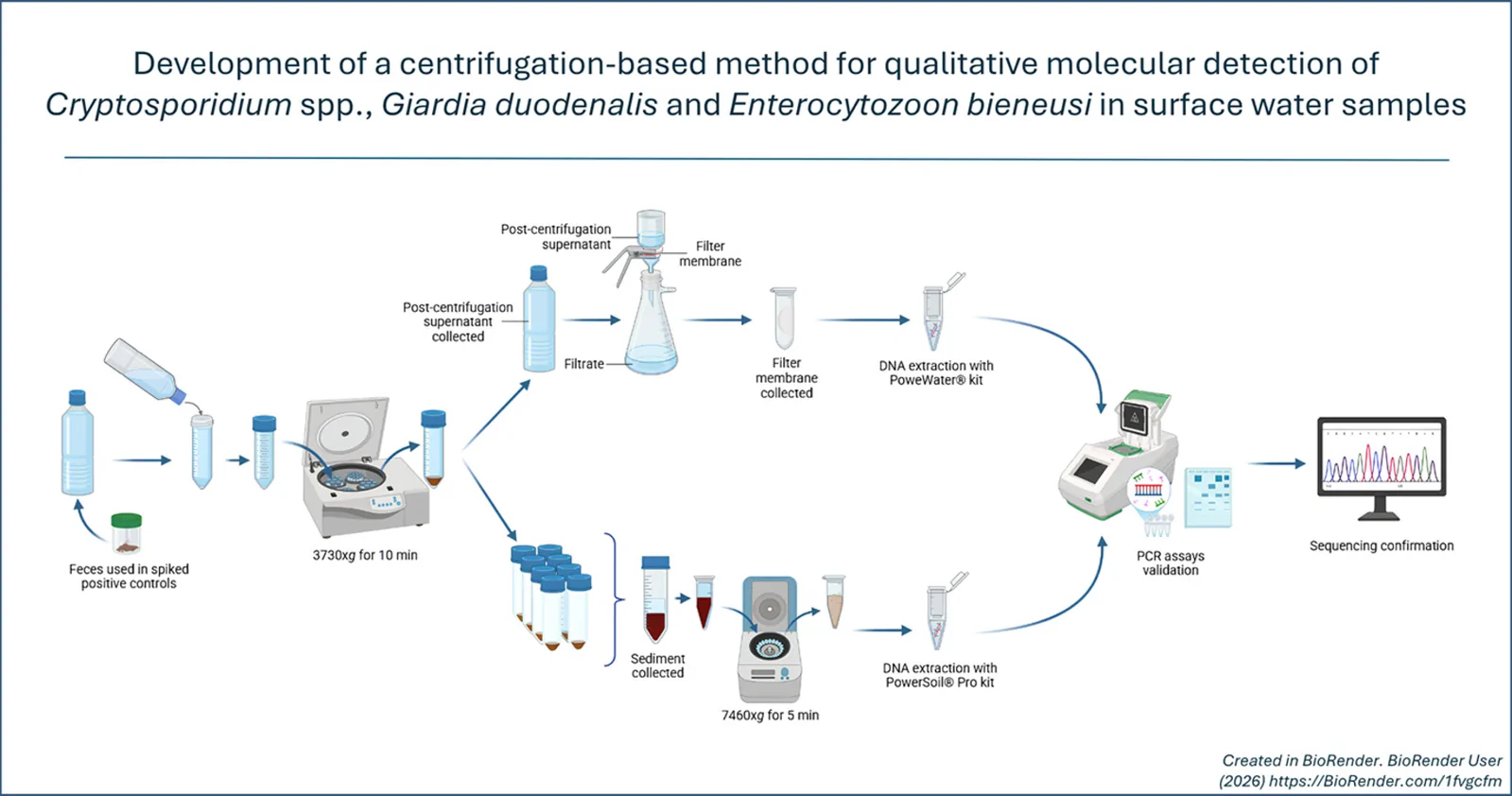

Specifications table

**Table:** 

Subject area	Veterinary Science and Veterinary Medicine
More specific subject area	Molecular parasitology; Waterborne parasites; Protozoa and Microsporidia.
Name of your method	Centrifugation-based method for multi-parasite DNA detection from surface water
Name and reference of original method	Not applicable
Resource availability	Extraction kit: https://www.qiagen.com/us/products/discovery-and-translational-research/dna-rna-purification/dna-purification/microbial-dna/dneasy-powerwater-kit?catno=14900–100-NF; https://www.qiagen.com/us/products/discovery-and-translational-research/dna-rna-purification/dna-purification/microbial-dna/dneasy-powersoil-pro-kit?catno=47014 Reagents: https://www.thermofisher.com/order/catalog/product/EP0714; https://www.thermofisher.com/order/catalog/product/R0241 Purification kit: https://ita.promega.com/en/products/nucleic-acid-extraction/clean-up-and-concentration/wizard-sv-gel-and-pcr-clean-up-system/?catNum=A9281 Filtration apparatus:https://shop.exactalabcenter.it/qsrch.htm?UID=55006a462922000000000000&KeyVal1=apparato+di+filtrazione Filter membranes: https://www.sigmaaldrich.com/IT/it/product/aldrich/wha1004047; https://www.sigmaaldrich.com/IT/it/product/aldrich/wha1006070; https://www.sigmaaldrich.com/IT/it/product/aldrich/wha1001047.

## Background

Zoonotic protozoa such as *Cryptosporidium* spp. and *Giardia duodenalis*, as well as microsporidia, such as *Enterocytozoon bieneusi*, are important waterborne pathogens in both public and veterinary health [1]. These parasites can infect humans and a broad range of animal hosts, including companion, livestock, and wild animals. Their transmissive stages are environmentally resistant and can persist in freshwater, contributing to their spread [2,3]. *Cryptosporidium* spp. and *G. duodenalis* have been frequently associated with waterborne outbreaks, highlighting the importance of monitoring these pathogens in aquatic matrices [4]. Although *E. bieneusi* is less commonly associated with outbreaks, its zoonotic potential and occurrence in water sources highlight the importance of including this parasite in environmental surveillance programs [5]. The current reference methodology for water analysis is mainly designed for isolation and enumeration of *Cryptosporidium* oocysts and *Giardia* cysts using filtration and specific immunomagnetic separation (IMS) extraction kits, and do not include microsporidia [6,7]. Filtration represents a valid approach for processing large volumes of water. However, it may be expensive and time-consuming, particularly for turbid waters, where frequent filter clogging can occur. Furthermore, more advanced filtration systems designed to process large water volumes without the use of vacuum pumps and cellulose filters may involve higher costs, and their performance may vary depending on the sample matrix. Overall, although widely used, these workflows remain labour-intensive, require specialised expertise and expensive equipment, and often rely on the processing of large water volumes [8]. In addition, they provide limited support for the integrated detection of different pathogens from the same water sample. Therefore, simplified and cost-effective methods are needed to process complex surface water matrices, particularly when based on lower sample volumes that still allow the detection of amplifiable DNA suitable for investigating multiple parasites presence within a single analytical pipeline. Such methods could be particularly useful for environmental surveillance in areas where routine monitoring requires a balance between analytical performance, cost-effectiveness and operational feasibility.

Here, we describe a centrifugation-based workflow for processing 10 litres of surface water, with the aim of using the pellet fraction as the primary matrix for parasite detection. To this purpose, a qualitative molecular approach for the detection of *Cryptosporidium* spp., *G. duodenalis*, and *E. bieneusi* from a single pellet sample through a streamlined protocol was developed and optimised. This method is designed to support the environmental surveillance of waterborne parasites in freshwater ecosystems by providing presence/absence results for the target organisms.

## Method details

### Positive - process controls

Positive – process controls were used to verify that DNA from each investigated parasite could be detected in the pellet after the entire sample-processing.

Three positive - process controls were prepared by homogenising 2 g of animal faeces in 2 L of environmental water. Faecal samples previously analysed in the laboratory and confirmed positive for target parasites were used as source material. Specifically, *G. duodenalis*-positive cat faeces, *Cryptosporidium* spp.-positive calf faeces, and *E. bieneusi*-positive pig faeces were used. To preserve the physicochemical characteristics of the study matrix, all spiking experiments were performed twice using surface water collected from one of the four sampling sites detailed below. Each control sample was processed separately according to the workflow described below.

### Study area and sample collection

To assess the applicability of the proposed method under field conditions, the study was conducted in an area located in northern Italy characterised by a dense hydrographic network and intensive livestock farming. Four sampling sites were selected to represent distinct surface water bodies defined by different hydrological and environmental conditions: the lake, the river upstream (inlet), and the downstream (outlet), and an irrigation canal, located in the Mincio river hydrographic basin (Italy). At each sampling event, water temperature ( °C), electrical conductivity corrected to 20 °C (µS cm⁻¹), pH, oxidation–reduction potential (ORP; mV), turbidity (FNU), dissolved oxygen (mg L⁻¹) and oxygen saturation (%) were measured using a portable multiparameter probe (HI98594, Hanna Instruments, Woonsocket, RI, USA). Total suspended solids were not measured.

Ten litres of surface water were collected monthly over a two-month period (November-December 2025) from each sampling site. Water samples were collected in the morning in sterile containers previously washed with sodium hypochlorite solution. After collection, samples were transported to the laboratory under refrigerated conditions (+4 °C) and protected from light to prevent degradation of biological material. Upon arrival, samples were stored at +4 °C until processing. All samples were processed within 24–48 h after collection. Samples from the different sites were processed sequentially. Pellet recovery and filtration of the corresponding supernatant were carried out consecutively on the same day. To optimise the workflow, centrifugation of the sample from the subsequent site was initiated concurrently with the filtration of the preceding sample.

### Pellet samples preparation, material concentration and DNA extraction

Each water sample (2 L spiked samples: n = 6; 10 L environmental samples: n = 8) was processed. Samples were aliquoted from containers by pouring water into sterile 50 mL tubes and then centrifuged for 10 min at 3730x*g,* allowing the separation of the sediment fraction from the supernatant. The use of two centrifuges operating simultaneously was chosen to reduce the time of processing (RWD M1416R, RWD Life Science Co., Ltd., Shenzhen, China; HERMLE Z 366, Hermle Labortechnik GmbH, Wehingen, Germany).

Using two centrifuges, each with 16-tubes capacity, a total volume of 1.6 L of water was processed every ten minutes (32 tubes per 10 min). A total of 48 sterile 50 mL tubes, arranged in three sets of 16, were used per each environmental sample, while processing each 2-L positive control required 32 sterile 50 mL tubes. A single batch of tubes was used exclusively for successive aliquots from the same water sample.

At the end of each centrifugation, the supernatant was collected and pooled, avoiding the resuspension of the obtained pellet. During this step, 1–3 mL of supernatant was retained in each 50 ml tube to facilitate homogenisation and recovery of the pellet, whereas the whole supernatant fraction was pooled in a sterile container, maintained at 4 °C and filtered later on the same processing day. Unloading, removal of the supernatant and refilling required approximately 5–6 min of hands-on work for each completed centrifuge run. Because the two centrifuges were operated in parallel, handling of the tubes from one centrifuge was generally performed while the other centrifuge was running. Consequently, much of the hands-on work overlapped with the overall elapsed processing time.

Following the initial processing cycles, the obtained pellet was then transferred by pipetting to new 50 mL sterile tubes, one for each positive - process control or sampling site, and underwent an additional centrifugation step under the same conditions described above. A small volume (1–3 mL) of supernatant was kept to resuspend the pellet and then transferred to 1.5 mL microcentrifuge tubes. Pellet samples were then stored at −80 °C, and DNA extraction was performed within two weeks of sample processing. Including initial aliquoting, repeated centrifugation and tube handling, pooling of the recovered sediment and final pellet concentration, the overall processing time was approximately 2 h per 10 L sample.

Before DNA extraction, the recovered pellet material was further concentrated by centrifugation for 5 min at 7460x*g*. The supernatant was then carefully removed by pipetting, leaving a highly compact pellet. DNA extraction was performed using the PowerSoil® Pro DNA Isolation Kit (QIAGEN, Hilden, Germany), with a minor modification to the initial lysis step. Briefly, PowerSoil® Pro C1 lysis buffer was added directly to the compact pellet to facilitate its homogenisation and transfer into the bead tubes supplied with the kit, minimizing material loss. Subsequent extraction steps were performed according to the manufacturer’s instructions.

DNA concentration and purity were then assessed using a spectrophotometer (NanoReady, LifeReal Biotechnology Co., Ltd., Hangzhou, China) (Table 1).

**Table 1:** Total DNA yield and quality ratios (A_260/280_ and A_260/230_) of filter membranes and pellet samples. Values represent the mean of two independent sampling campaigns, with individual results for each campaign reported in brackets in ascending order (including blanks, lake, river inlet/outlet, and canal).

Sample	Type of sample^1^	Pore size (µm)	Total DNA yield (ng/μL)	DNA purity	
				A_260/280_ ratio	A_260/230_ ratio
*Cryptosporidium* sp. positive control	F	20–25	1.28 (0.86 – 1.7)	1.17 (0.48 – 1.85)	0.32 (0.03 – 0.29)
		11	3.01 (0.72 – 5.3)	1.04 (0.33 – 1.74)	0.05 (0.01 – 0.08)
		3	1.42 (1.14 – 1.7)	0.52 (0.43 – 0.61)	0.04 (0.03 −0.05)
	P	-	224.1 (219.64 - 228.47)	2.03 (2.03 - 2.03)	1.65 (1.55 - 1.75)
*Giardia duodenalis* positive control	F	20–25	1.14 (1.03 - 1.24)	0.61 (0.52 - 0.7)	0.21 (0.07 - 0.14)
		11	1.87 (0.9 - 2.83)	1.07 (0.58 - 1.55)	0.02 (0.00 – 0.04)
		3	0.86 (0.72 – 1)	0.33 (0.31 - 0.35)	0.07 (0.05 −0.08)
	P	-	387.96 (311.96 – 463.96)	2.06 (2.05 – 2.06)	1.21 (0.76 - 1.66)
*Enterocytozoon bieneusi* positive control	F	20–25	2,23 (1.45- 3)	1.98 (1.05 – 2.9)	0.01 (0.00 - 0.02)
		11	3.79 (1.2 - 6.37)	0.73 (0.41 - 1.05)	0.02 (0.01 - 0.02)
		3	3.27 (1.74 – 4.8)	3.04 (1.26 - 1.78)	0.07 (0.04 - 0.09)
	P	-	324.3 (372.84 – 275.83)	2.05 (2.04 - 2.05)	1.68 (1.38 – 1.97)
Blank filters	F	20–25	3.86 (1.82 – 5.9)	1.77 (1.46 – 2.08)	0.11 (0.07 – 0.15)
		11	3.27 (0.63 – 5.9)	1.33 (0.97 – 1.68)	0.03 (0.02 – 0.03)
		3	4.95 (0.49 – 9.4)	1.18 (0.66 – 1.69)	0.32 (0.21 – 0.43)
Lake	F	20–25	3.6 (2.05 - 5.2)	1.58 (1.03 - 2.14)	0.23 (0.3 - 0.32)
		11	5.38 (4.21 – 6.55)	1.46 (1.04 – 1.87)	0.23 (0.12 – 0.34)
		3	5.42 (2.13 – 8.7)	1.20 (0.96 – 1.43)	0.06 (0.05 – 0.07)
	P	-	479.49 (213.87 – 745.1)	1.96 (1.9 – 2.01)	1.27 (1.17 – 1.36)
River (inlet)	F	20–25	7.8 (1.81 - 13.8)	2.05 (1.41 - 2.7)	0.18 (0.03 - 0.34)
		11	7 (2.84 – 11.15)	1.57 (1.13 – 2)	0.49 (0.25 – 0.73)
		3	5.16 (1.71 – 8.6)	1.6 (1.17 – 2.03)	0.16 (0.03 – 0.28)
	P	-	231.21 (135.91 – 326.5)	1.96 (1.9 – 2.01)	1.02(0.56 – 1.48)
River (outlet)	F	20–25	5.7 (5.19 - 6.3)	1.94 (1.37 - 2.51)	0.1 (0.01 - 0.18)
		11	4.94 (4.43 – 5.45)	1.74 (1 – 2.47)	0.17 (0.16 – 0.17)
		3	3.52 (0.84 – 6.2)	1.34 (0.5 – 2.18)	0.3 (0.1 – 0.5)
	P	-	529.69 (368.27 – 691.1)	1.96 (1.91 – 2.01)	1.42 (0.97 – 1.86)
Canal	F	20–25	6 (0.37 - 11.7)	1.09 (0.31 - 1.87)	0.36 (0.02 - 0.69)
		11	4.57 (1.14 – 8)	1.77 (0.64 – 2.89)	0.09 (0.03 - 0.14)
		3	3.74 (0.27 – 7.2)	1.06 (0.16 – 1.95)	0.18 (0.03 – 0.33)
	P	-	418.83 (295.65 – 542)	1.94 (1.88 – 2)	0.79 (0.61 – 0.97)

^1^ F = filter membranes; P = pellets.

### Water samples filtration and DNA extraction from filter membrane

Supernatant filtrations were performed using a Millipore® vacuum filtration apparatus (Merck KGaA, Darmstadt, Germany), equipped with a filtration unit connected to a vacuum pump. Whatman® cellulose membrane filters with a diameter of 47 mm were employed. To remove larger particles and prevent rapid clogging, samples were first filtered through Whatman® grade 4 filters (pore size: 20–25 µm) (Cytiva, Marlborough, MA, USA). The resulting filtrates were subsequently processed using Whatman® grade 1 (pore size: 11 µm) and grade 6 filters (pore size: 3 µm). All membrane grades, including the 20–25-µm prefilters, were retained and processed separately for DNA extraction, and the DNA obtained from each membrane fraction was tested for all three investigated parasites. The selected pore sizes were considered in relation to the approximate dimensions of *G. duodenalis* cysts (∼8 × 10 µm) [9], *Cryptosporidium* spp. oocysts (∼4 × 6 µm) and *E. bieneusi* spores (∼0.7 × 1.08 µm) [2,10]. It should be noted that *E. bieneusi* spores are smaller than the 3 µm pore size of the membrane filter employed; therefore, their retention by the filter may have been limited, potentially reducing spore recovery. However, the use of membranes with smaller pore sizes was not feasible due to the turbidity of the water samples, which would have caused rapid filter clogging.

One litre of distilled water was filtered through each membrane type as blanks to assess possible contamination from the filtration apparatus. In this study, environmental water samples exhibited varying degrees of turbidity, which affected the filtration process. Specifically, the canal sample showed the highest turbidity and, together with the lake sample, required the use of more than one filter for each pore size (specifically, up to 3 filter membranes were used for each grade). After filtration, membranes were stored at −80 °C until DNA extraction, which was performed within two weeks of sample processing.

DNA extraction from filter membranes was performed using PowerWater® DNA Isolation Kit (QIAGEN, Hilden, Germany). Filters with the same pore size obtained from the same water sample were pooled and processed as a single sample for DNA extraction. Total DNA concentration and purity were assessed using a spectrophotometer (NanoReady, LifeReal Biotechnology Co., Ltd., Hangzhou, China) (Table 1).

A schematic representation of the proposed water sample-processing workflow is provided in Supplementary Material S1.

## Method validation

### Assessment of DNA detection and PCR inhibition

Given that ciliates are ubiquitous protozoa widely distributed in terrestrial and aquatic ecosystems, their detection can be used as an indicator of amplifiable eukaryotic DNA in environmental samples [11]. Accordingly, prior to specific parasite detection, a qualitative PCR targeting a region of the Ciliophora 18S rRNA gene was performed as described by [12].

Cycling conditions were as follows: initial denaturation at 95 °C for 3 min, followed by 40 cycles of denaturation at 95 °C for 30 s, annealing at 50 °C for 30 s and extension at 72 °C for 30 s, with a final extension at 72 °C for 5 min.

Each reaction was performed in a final volume of 20 μL containing 2 μL of DNA template, 1X DreamTaq® Reaction Buffer (Thermo Fisher Scientific, Waltham, MA, USA), 0.2 mM dNTPs, 1 μM of each primer, 0.5 U DreamTaq® DNA Polymerase and DNase-free water. Dilutions of pellet DNA (1:10) were also tested to reduce the potential effect of PCR inhibitors or excessive template concentration. No template controls (NTCs) and positive controls, consisting of previously sequenced ciliate DNA samples, and filtration -process blanks were included.

### PCR assays validation on spiked positive controls and samples

Target- specific qualitative PCR assays were performed to assess the ability of the proposed workflow to recover amplifiable DNA from each of the three different parasites.

For all three parasite-specific PCRs, DNA extracted from pellet samples was tested both undiluted and after a 1:10 dilution in nuclease-free water. Each template concentration was analysed in three independent nested-PCR replicates. A sample was considered PCR-positive when at least one of the three replicates produced an amplicon of the expected size. NTCs and filtration-process blanks were tested in triplicate.

*Cryptosporidium* spp. was detected by a nested PCR targeting a 214 bp fragment of the 18S rRNA gene, using primers and cycling conditions previously described by [13]. Each reaction was performed in a final volume of 20 μL containing 1X DreamTaq® Reaction Buffer (Thermo Fisher Scientific, Waltham, MA, USA), 0.2 mM dNTPs, 500 nM of each primer, 0.5 U DreamTaq® DNA Polymerase and DNase-free water. The primary PCR was carried out using 3 μL of DNA template, while 2 μL of the primary PCR product was used as template for the secondary PCR.

Regarding *Giardia duodenalis,* a nested PCR targeting a 530 bp fragment of the triose phosphate isomerase (*tpi*) gene was carried out following previously described primers and cycling conditions [14], with a minor modification to the thermal protocol consisting of 40 cycles for both PCR rounds. PCR reactions were conducted in 20 μL containing 1X DreamTaq® Reaction Buffer (Thermo Fisher Scientific, Waltham, MA, USA), 0.2 mM dNTPs, 500 nM of each primer, 0.5 U DreamTaq® DNA Polymerase and DNase-free water. For the first amplification round, 2 μL of DNA sample were added to the reaction mixture; for the second round, 2 μL of the primary PCR amplicon were used as template.

*Enterocytozoon bieneusi* was detected by nested PCR targeting the ITS region, yielding an approximately 390 bp fragment, using primers previously described by [15]. Each reaction was performed in final volume of 20 μL containing 1X DreamTaq® Reaction Buffer (Thermo Fisher Scientific, Waltham, MA, USA), 0.2 mM dNTPs, 1 μM of each primer, 0.5 U DreamTaq® DNA Polymerase and DNase-free water. The primary PCR was carried out using 3 μL of DNA template, while the secondary PCR was performed using 1 μL of the primary PCR product as template. Primary PCR cycling conditions were as follows: initial denaturation at 94 °C for 5 min, followed by 35 cycles at 94 °C for 45 s, 57 °C for 45 s and 72 °C for 1 min, with a final extension at 72 °C for 10 min. The secondary PCR was run under the same conditions, except that the annealing temperature was set at 55 °C and the number of cycles was reduced to 30.

All PCR products were visualised by agarose gel electrophoresis after staining with ethidium bromide. Bands corresponding to amplicons of the expected molecular size were excised from the gel and purified using Wizard® SV Gel and PCR Clean-Up System kit (Promega, Madison, WI, USA) according to the manufacturer’s instructions. Purified PCR products were submitted to Eurofins Genomics (Ebersberg, Germany) for Sanger sequencing, and the obtained sequences were analysed using BioEdit and compared with sequences available in GenBank® using BLAST [ 16]; https://blast.ncbi.nlm.nih.gov).

The following results were obtained:

I) Physicochemical characteristics recorded with the multiparameter probe highlighted environmental water differences among sampling sites (Table S2).
II) No amplification was observed in any of the three replicates of the no-template controls or filtration-process blanks in either the ciliate or parasite-specific PCR assays.
III) In the positive - process control, the proposed centrifugation-based workflow allowed the concentration of the target parasites in the pellet fraction. Notably, *Cryptosporidium* spp., *G. duodenalis* and *E. bieneusi* were successfully amplified from the recovered pellets, whereas for filter samples of *G. duodenalis* and *E. bieneusi* no specific amplification was obtained. A weak PCR amplification was observed in the *Cryptosporidium* filter fraction; however, the yield of PCR product was insufficient for sequencing, and its specificity could not be confirmed.
IV) In environmental samples, PCR on ciliates showed amplification in all diluted and undiluted DNA from pellets (8/8), confirming the amplifiability of the sample. In contrast, amplification from filter membranes was less consistent (4/8).
V) Target-specific PCR assays revealed the presence of at least one target parasite in all pellet samples, whereas all corresponding filter membranes were negative. Overall, *E. bieneusi, G. duodenalis* and *Cryptosporidium* spp. were detected in 8/8, 7/8 and 6/8 pellet fractions, respectively. Two expected-size bands obtained from filter membranes for *G. duodenalis* were not confirmed by sequencing and were therefore considered non-specific. Detailed molecular results are presented in Table 2, while BLAST results for successfully sequenced amplicons are provided in Supplementary Table S3.

**Table 2:** Detection of target parasites in sediment/pellet fractions and filter membranes from environmental water samples collected during two sampling campaigns.

Target parasite	Sampling campaign	Sampling site	Result of PCR^§^	
			Filter membranes°	Pellet
Ciliate	1st	Lake	0	1
		River (inlet)	1	1
		River (outlet)	0	1
		Canal	1	1
	2nd	Lake	0	1
		River (inlet)	1	1
		River (outlet)	1	1
		Canal	0	1
	Overall positivity		4	8
*Cryptosporidium* spp.	1st	Lake	0	0
		River (inlet)	0	0
		River (outlet)	0	1
		Canal	0	1
	2nd	Lake	0	1
		River (inlet)	0	1
		River (outlet)	0	1
		Canal	0	1
	Overall positivity		0	6
*Giardia duodenalis*	1st	Lake	0	0
		River (inlet)	0*	1
		River (outlet)	0	1
		Canal	0*	1
	2nd	Lake	0	1
		River (inlet)	0	0
		River (outlet)	0	1
		Canal	0	1
	Overall positivity		0	6
*Enterocytozoon bieneusi*	1st	Lake	0	1
		River (inlet)	0	1
		River (outlet)	0	1
		Canal	0	1
	2nd	Lake	0	1
		River (inlet)	0	1
		River (outlet)	0	1
		Canal	0	1
	Overall positivity		0	8

PCR results for *Cryptosporidium* spp., *G. duodenalis* and *E. bieneusi* are reported for each sampling site and matrix fraction.

* Expected-size band with non-specific amplicon by sequencing (Expected-size bands were obtained from the 11-µm filter membrane DNA sample for the River (inlet) and from both the 11- and 3-µm membrane DNA samples for the Canal sample). **^§^** 0= negative, 1=positive. ° Results refer to all pore size membranes (20–25, 11 and 3 μm).

These findings suggest that this centrifugation-based method may represent a practical alternative to filtration for the molecular surveillance of waterborne parasites, particularly when membrane clogging limits sample processing. The number of samples analysed in the present study was limited, as this workflow is currently being applied within an ongoing one year-long monitoring project.

In conclusion, the proposed pellet-based workflow offers several practical advantages for the molecular surveillance of waterborne parasites investigated. First, it relies on standard laboratory equipment and does not require dedicated filtration devices, vacuum pumps and filter membranes. This makes the procedure more accessible and less time-consuming, especially for routine screening or resource-limited settings. Second, direct processing of the sediment fraction reduces sample fragmentation and allows the simultaneous detection of different parasite taxa from the same water sample. In contrast, filtration-based approaches may require different filter pore sizes according to the dimension of the target parasite infective stages. This can lead to the generation of multiple fractions from the same water sample, each requiring separate processing and DNA extraction. Third, this approach eliminates problems related to membrane clogging, which can limit the processing of turbid environmental waters. Moreover, DNA yields in filter membranes used to process the post-centrifugation supernatants of environmental samples were comparable to those measured in blank filters, suggesting a background noise of the extraction procedure rather than residual biological components. However, four environmental filter fractions tested positive in the ciliate control assay, confirming the presence of few DNA molecules in the supernatant fraction. In contrast, no amplification was detected in all blank filters. Finally, the pellet fraction enabled consistent molecular detection of target-parasites DNA, supporting its use as an informative matrix for PCR-based analysis. However, these findings are limited to the experimental conditions evaluated. Since the filter fractions were obtained from post-centrifugation supernatants, the present study does not demonstrate improved recovery, or the replacement of direct filtration or established reference methods.

A practical comparison between the pellet-based workflow and filtration-based processing is provided in Table 3.

**Table 3:** Comparison and evaluation of costs and processing time between pellet-based and filtration-based workflows evaluated in this study.

Feature	Pellet-based centrifugation	Filtration-based workflow
Reusable equipment	Centrifuge	Vacuum pump and filtration apparatus
Per-sample consumables	50 mL centrifuge tubes, 1.5 mL tubes, pasteur pipettes.	Filter membranes, sterile tubes.
Sterility between samples	Mainly sterile disposable tubes and pipettes	Filtration apparatus must be sterilized between samples
Number of fractions	One pellet fraction	Potentially multiple filter fractions
DNA extraction kit	PowerSoil® Pro kit Total number of reactions: 11	PowerWater® Total number of reactions: 39
Estimated cost without extraction	∼€10–20/sample	∼€25–50/sample
Estimated cost with extraction	∼€25–30/sample	∼€45–80/sample
Main practical limitation	Requires repeated centrifugation steps	Clogging and cleaning/sterilization between samples
Multi-target screening	Same DNA extract can be used for multiple parasite taxa	Different filters/pore sizes may be needed for different targets
Processing time	∼2–2.5 h per 10 L sample	Variable, depending on filtration speed, clogging and the number of filters with different pore sizes. For turbid waters, pre-filtration through larger-pore filters may be required to prevent early clogging; in our experience, processing required ∼3.5–4 h per 10 L sample.

### Limitations

This method was developed to enable the detection of DNA from *Cryptosporidium* spp., *G. duodenalis*, and *E. bieneusi* within a single surface-water sample, avoiding the filtration step which may be time-consuming and affected by rapid membrane clogging specially in turbid samples. Although reliable results were obtained in this study and the sample processing was cost-effective, some limitations should be acknowledged.

First, processing large water volumes requires multiple centrifugation steps, making the workflow relatively time-consuming. However, highly turbid samples often require pre-filtration centrifugation to reduce membrane clogging and improve sample handling, meaning that combined centrifugation–filtration approaches may be even more time-consuming.

Second, although the sampled waters were characterised for temperature, conductivity, pH, oxidation–reduction potential, turbidity, dissolved oxygen, oxygen saturation and chlorophyll-a, total suspended solids, particle-size distribution, dissolved organic matter and metal concentrations were not measured. Therefore, the potential influence of these unmeasured matrix characteristics on sedimentation efficiency, inhibitor co-concentration and molecular detection could not be fully evaluated.

In addition, the comparison between the sediment pellet and membrane-filtered fractions was based exclusively on qualitative presence/absence PCR results. Analytical sensitivity, limit of detection, and parasite recovery efficiency were not quantitatively assessed using known parasite concentrations. Consequently, the higher detection frequency observed in sediment pellets under the tested conditions should not be interpreted as evidence of greater analytical sensitivity.

An additional consideration concerns the detection of *E. bieneusi* in the membrane-filtered fraction. Since its spores are extremely small (∼0.7 × 1.08 μm), if present in the water sample they may pass through 3-μm membrane pore size, therefore remaining undetected when only filtration is performed [2]. Filters with a smaller pore size, such as 0.2 μm, could improve spore retention. However, when these membranes were tested, they rapidly clogged during filtration of environmental samples and were unsuitable for processing 10 L of surface water. Their use would require substantially longer filtration times, multiple membranes and higher costs. Therefore, the 3-μm membrane was selected as a practical compromise, although it was not expected to efficiently retain *E. bieneusi* spores. Consequently, the absence of confirmed target-specific amplification in the filter fractions cannot be interpreted as evidence that pellet processing was more efficient than filtration for this organism. Detection in the pellet samples only demonstrates that the centrifugation-based workflow enabled qualitative molecular detection of *E. bieneusi* under the conditions tested.

Further limitations concern the study design. The filter fractions were obtained from post-centrifugation supernatants rather than from independently processed aliquots, and the field evaluation included only eight samples from four sites within a single hydrographic basin over two consecutive months. Moreover, the parasite load in the spiking material was not quantified.

Finally, different parasites may distribute differently between pelleted and filtered fractions, and recovery may vary according to the target organism and the characteristics of the water matrix. Since the present study focused exclusively on *Cryptosporidium* spp., *G. duodenalis* and *E. bieneusi*, and was evaluated using a defined panel of surface-water samples and matrix-matched spiked controls, the applicability of this pellet-based approach to other parasite taxa remains to be fully established. Before extending the method to additional targets, their recovery in the pellet fraction should be verified, while the corresponding supernatant or filter fraction should also be analysed during validation. Only after confirming consistent detection of the target organisms DNA in the pellet fraction, filtration could be excluded from the workflow.

## CRediT author statement

**Susanna Adelaide Sechi**: Methodology, Validation, Investigation, Writing - Original Draft, **Chiara Stocchero**: Methodology, Validation, Investigation, Writing - Original Draft, **Carolina Allievi**: Methodology, Validation, Writing - Review & Editing, **Diego Copetti**: Conceptualization, Resources, Project administration, Funding acquisition, Writing - Review & Editing, **Fabrizio Stefani**: Conceptualization, Resources, Writing - Review & Editing, **Lucia Valsecchi**: Conceptualization, Writing - Review & Editing, **Alessandra Cafiso**: Methodology, Writing - Review & Editing, **Chiara Bazzocchi**: Conceptualization, Methodology, Validation, Writing - Review & Editing, **Maria Teresa Manfredi**: Conceptualization, Methodology, Resources, Writing - Review & Editing, Supervision, Project administration, Funding acquisition

## Supplementary material *and/or* additional information [OPTIONAL]

S1. Schematic workflow of the proposed water sample-processing method.

## Declaration of competing interest

The authors declare that they have no known competing financial interests or personal relationships that could have appeared to influence the work reported in this paper.

## Data Availability

Data will be made available on request.
