# Supplementary material for: Development of a centrifugation-based method for the qualitative molecular detection of Cryptosporidium spp., Giardia duodenalis and Enterocytozoon bieneusi in surface water samples
Source: MethodsX. 2026 Oct 8;17:104195. doi: 10.1016/j.mex.2026.104195 (PMC13682023; doi:10.1016/j.mex.2026.104195)
Supplement: Supplementary file 2 [file mmc2.docx]

| **Sampling campaign** | **Sampling site** | **Temperature (°C)** | **Conductivity (µS cm⁻¹)** | **pH** | **ORP (mV)** | **Turbidity (FNU)** | **Dissolved oxygen (mg L⁻¹)** | **Oxygen saturation (%)** |
| --- | --- | --- | --- | --- | --- | --- | --- | --- |
| 1st | River upstream (inlet) | 12,22 | 393 | 7,93 | 211,6 | 5,5 | 11,12 | 97,1 |
| 1st | Irrigation canal | 9,52 | 912 | 8,01 | 263,0 | 60,1 | 11,56 | 95,0 |
| 1st | Lake | 12,03 | 452 | 7,83 | 270,3 | 7,1 | 9,00 | 83,2 |
| 1st | River downstream (outlet) | 13,04 | 483 | 7,81 | 263,1 | 20,0 | 9,44 | 80,2 |
| 2nd | River upstream (inlet) | 9,65 | 358 | 8,04 | 243,2 | 0,8 | 11,12 | 97,1 |
| 2nd | Irrigation canal | 7,03 | 920 | 8,10 | 242,7 | 17,5 | 11,56 | 95,0 |
| 2nd | Lake | 8,11 | 499 | 7,89 | 260,5 | 6,4 | 9,00 | 83,2 |
| 2nd | River downstream (outlet) | 8,61 | 538 | 7,80 | 269,0 | 15,5 | 9,44 | 80,2 |

**Table S2. Physicochemical characteristics of the surface-water samples collected from the four sampling sites during the two sampling campaigns.**

Abbreviations: ORP, oxidation–reduction potential; FNU, Formazin Nephelometric Units.

Conductivity values were corrected to 20 °C.
